# Hyams Grade and Ki‐67 as Predictive Factors for Primary Treatment Failure in Olfactory Neuroblastoma

**DOI:** 10.1002/lary.32238

**Published:** 2025-05-09

**Authors:** Michael Koch, Mathias Balk, Sven Schlaffer, Abbas Agaimy, Heinrich Iro, Sarina Müller

**Affiliations:** ^1^ Department of Otorhinolaryngology and Head and Neck Surgery University of Erlangen–Nuremberg Erlangen Germany; ^2^ Department of Neurosurgery University of Erlangen–Nuremberg Erlangen Germany; ^3^ Institute of Pathology University of Erlangen–Nuremberg Erlangen Germany

**Keywords:** esthesioneuroblastoma, failure, olfactory neuroblastoma, prognosis, progression, recurrence, therapy, tumor

## Abstract

**Background/Objective:**

Tumor progression and first recurrence (TPR) after curative treatment for olfactory neuroblastoma can be regarded as primary treatment failure. Prognostic parameters for TPR and primary tumor‐progression‐free and recurrence‐free survival (TPRFS) have not been sufficiently investigated in the literature.

**Methods:**

Data for 43 patients were analyzed retrospectively to evaluate prognostic parameters for TPR after curative treatment for olfactory neuroblastoma: age, age < / ≥ 50 years, sex, tumor classifications, curative therapy (monotherapy vs. combined, R0 vs. R1/2/x resection), Hyams‐grade (grades, grade I–II/III–IV), and Ki‐67 labeling index (values, labeling index < / ≥ 10%). The primary endpoints were TPR and TPRFS. Parameters that were significant after univariate analysis and Kaplan–Meier survival analysis were included in multiple regression and Cox regression analysis.

**Results:**

After univariate analysis, younger age (*p* = 0.032) and higher Ki‐67 values (*p* = 0.001) were significantly negatively associated with time to the development of TPR. TPRFS according to Kaplan–Meier was significantly poorer with Hyams‐grade III–IV (*p* = 0.002) and Ki‐67 ≥ 10% (*p* = 0.001). After Cox regression analysis, TPRFS according to Kaplan–Meier was weekly significantly poorer for younger age (*p* = 0.033) and highly significantly worse for Hyams‐grade III–IV (*p* = 0.005) and a Ki‐67 LI ≥ 10% (*p* = 0.009). Tumor‐stage classifications and all therapeutical parameters were not significantly associated with TPRFS.

**Conclusions:**

Out of a panel of parameters tested, younger age, Hyams‐grade III–IV, and a Ki‐67 LI ≥ 10% were significantly associated with a significantly worse TPRFS after multivariate Cox regression analysis. In particular, parameters such as Hyams‐grade and the Ki‐67 LI should be included in management considerations in olfactory neuroblastoma at an early stage.

**Level of Evidence:**

3

## Graphical abstract

The aim of the present study was to investigate the influence of various prognostic parameters on the occurrence of primary tumor progression and/or tumor recurrence (TPR) in esthesioneuroblastoma after primary curative therapy in a single center. Time to first TPR in esthesioneuroblastoma was significantly shorter in cases with a younger age and higher Ki‐67 values. Poorer TPR‐free survival was significantly associated with Hyams‐grade III–IV and a Ki‐67 LI ≥ 10%.

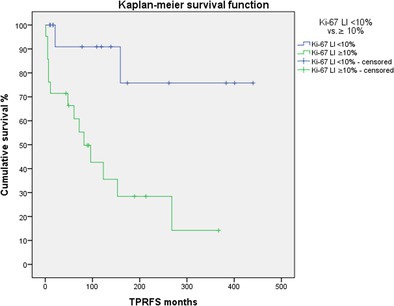

## 1 Introduction

Olfactory neuroblastoma (ONB) is a rare tumor with a relatively good long‐term prognosis [1, 2, 3, 4, 5, 6]. A multidisciplinary approach, including surgery with negative margins in combination with radiotherapy, is associated with the best prognosis. Chemotherapy appears to be indicated in advanced tumor stage, particularly in pediatric ONB [4, 5, 7, 8, 9, 10, 11, 12, 13, 14, 15, 16, 17, 18, 19, 20, 21].

Known prognostic factors are age [9, 22, 23, 24, 25, 26], tumor stage/classification systems [27, 28, 29, 30, 31, 32], histopathologic factors like Hyams grading [11, 23, 28, 33, 34, 35, 36, 37, 38, 39, 40, 41, 42, 43, 44, 45] or the Ki‐67 labeling index (Ki‐67 LI) [46, 47, 48] and therapy‐related factors [4, 5, 7, 8, 9, 10, 11, 12, 13, 14, 15, 16, 17, 18, 19, 20, 21].

Although the prognostic significance of most tumor classification systems has been confirmed, also in comparison with each other [4, 11, 13, 19, 22, 34, 45, 49], modified classifications with inclusion of additional factors have been proposed due to some inadequacies [4, 9, 11, 13, 18, 22, 23, 25, 33, 34, 36, 42, 49, 50, 51], particularly Hyams grading and the Kadish classification in one report [52]. The prognostic impact of histopathologic parameters such as Hyams‐grading [11, 23, 28, 33, 34, 35, 36, 37, 38, 39, 40, 41, 42, 43, 44, 45] and the Ki‐67 LI has been reported [47, 48].

Development of primary tumor progression (TP) and first recurrence (TR) are the main criteria for primary treatment failure (tumor progression/recurrence, TPR). TP was associated with a significantly poorer outcome [29, 34, 53, 54, 55, 56, 57, 58], although a curative therapy has been performed.

Recurrent ONB has been assessed in many studies, mainly focusing on time‐dependency, the pattern, causes, and salvage therapy [3, 6, 34, 44, 56, 59, 60, 61, 62, 63, 64, 65, 66, 67, 68, 69]. Recurrences are significantly associated with an advanced tumor stage [45, 64, 66, 68, 70], with a reduced survival time [18, 45, 66, 69], and with the expression of biomarkers like Hyams grade (grade III–IV) [34, 44, 45, 66] or insulinoma‐associated protein 1 (INSM1) [68].

Prognostic factors associated with TPR in ONB were not specifically elaborated, even in population‐based [19, 22, 24, 26, 39] or hospital‐based studies [9, 10, 25, 71].

The aim of the present study was therefore to investigate the influence of the above‐mentioned prognostic parameters on the occurrence of TPR after primary curative therapy in a single center.

## 2 Methods

This retrospective study was conducted at the Department of Otorhinolaryngology, Head and Neck Surgery of Friedrich Alexander University Erlangen–Nuremberg. The department's database and clinical reports were searched for patients with first presentations in the department who had been treated with curative intent due to histologically confirmed ONB. The study was conducted in full accordance with ethical principles, including the World Medical Association Declaration of Helsinki (version 2002). Approval for the study was obtained from the local institutional review board of FAU Erlangen–Nuremberg (no. 20‐292‐Br).

Cases which had primary surgical resection with or without adjuvant therapy were included. The parameters investigated were sex (male/female ratio), age, age < 50 vs. ≥ 50 years, tumor classification systems (Kadish A–C/Morita A–D: categories A/B vs. C/D; Dulguerov&Calcaterra: categories T1/2 vs. T3/4; Koka: categories T1/2 vs. T3/4; Biller: categories T1 vs. T2 vs. T3 vs. T4 and T1 vs. T2–4; Resto: categories T1 vs. T2 vs. T3 and T1 vs. T2/3), N status, M status, Hyams‐grade (categories I–II vs. III–IV), Ki‐67 LI (categories < 10% vs. ≥ 10%), surgery (yes vs. no), surgical resection state (margin status: negative vs. unknown/positive), administration of radiotherapy (RT; yes vs. no, RT dosage) and therapy (monomodal vs. combined). Combined therapy was defined as surgery and RT with or without chemotherapy.

Follow‐up examination was done by the Department of ENT, Head and Neck surgery, the department of Neurosurgery, and, according to the involvement, also by the Department of Radio‐Oncology. Clinical endoscopic examination and imaging (ultrasound, CT‐scan, MRI) were performed according to the well standardized tumor surveillance schedule.

The end points of the study were primary treatment failure (TPR) despite curative therapy, and the impact of the prognostic parameters assessed on the Kaplan–Meier estimation of TPR‐free survival (TPRFS). The time between the end of the curative treatment to the first diagnosis of TPR was noted and used as TPRFS time and to calculate TPRFS by the Kaplan–Meier approach.

### 2.1 Statistical Analysis

SPSS Statistics for Windows, version 26, was used (IBM Corporation, Armonk, New York, USA). All data are given as mean ± SEM, median, and range. Bivariate correlations were calculated using the Pearson correlation coefficient. Differences and associations between the groups for categorical variables were calculated using the Pearson Chi‐square test. Differences and associations between groups for continuous variables were calculated using the Mann–Whitney *U* test. Time‐dependent event curves were calculated according to the Kaplan–Meier method. Comparison of different groups was conducted using the log‐rank test. Significant parameters after univariate analysis were chosen for multiple regression analysis. For the multivariate analysis, Cox regression was used for the analysis of time‐dependent events. The receiver operating characteristic (ROC) curve for Ki‐67 values (%) relative to the event of TPR was calculated. The significance level was *p* ≤ 0.05.

## 3 Results

### 3.1 Patient Data

Forty‐three patients were included. At first presentation, their mean age was 52.0 years; 67.4% had an age of ≥ 50, and 51.2% were female. As assessed by the various classification systems, advanced tumors were present in 41.9%–55.9% (Tables 1 and 2). Only one patient had a positive N‐status (2.2%, N3‐status), and none had distant metastases at first presentation.

**TABLE 1:** Tumor Stages in the Kadish, Morita, Biller, Dulguerov & Calcaterra, Koka, and Resto Classifications in 43 Patients with Olfactory Neuroblastoma at First Presentation.

Classification type	Kadish 1975 (A–C)	Morita 1993 (A–D)	Biller 1990 (T1–T4)	Dulguerov & Calcaterra 1992 (T1–T4)	Koka 1998 (T1–T4)	Resto 2000 (T1–T3)
Findings based on	Clinical + Radiologic	Clinical + Radiologic	Clinical + Surgical	Clinical + Radiologic	Clinical + Radiologic	Clinical + Surgical
TU‐stage						
A or T1 (%)	1 (2.3)	1 (2.3)	19 (44.2)	6 (14.0)	16 (37.2)	19 (44.2)
B or T2 (%)	23 (53.5)	26 (53.5)	22 (51.2)	19 (44.2)	5 (11.6)	22 (51.2)
C or T3 (%)	19 (44.2)	19 (44.2)	2 (4.7)	16 (37.2)	12 (27.9)	2 (4.7)
D or T4 (%)	—	0 (0)	0 (0)	2 (4.7)	10 (23.3)	—

**TABLE 2:** Demographic data, staging in the various tumor classification systems, histopathologic parameters, treatment and survival/outcome, including univariate and multivariate analyses, in all patients, patients without (*n* = 25) and with tumor progression/recurrence (*n* = 18) after curative surgery in 43 patients with olfactory neuroblastoma.

Patients/groups parameter	All cases (*n* = 43)	Cases without primary treatment failure (no TPR, *n* = 25)	Cases with primary treatment failure (TPR, *n* = 18)	Univariate analysis: Compare of groups ± TPR: Mann–Whitney *U* test; Kaplan–Meier estimation (Log‐rank‐test, *p*)	Multivariate analysis/Compare of groups ± TPR: Cox regression analysis (*p*)
Gender					
M	48.8% (21/43)	52.0% (13/25)	44.4% (8/18)	n.s. (*p* = 0.400)^d^	—
F	51.2% (22/43)	48.0% (12/25)	55.6% (10/18)		
Age (years)	52 ± 2.3 (M 52.0, R 15–84)	55.0 ± 3.3 (M 56.0, R 15–84)	48.1 ± 3.1 (M 47.0; R 27–77)	***p* = 0.032** ^a^	***p* = 0.033**
Age (years)					
< 50 (*n*, %)	32.6% (14/43)	20.0% (5/25)	50.0% (9/18)	n.s. (*p* = 0.299)^d^	—
≥ 50 (*n*, %)	67.4% (29/43)	80.0% (20/25)	50.0% (9/18)		
Mod. Kadish/Morita					
A/B	55.8% (24/43)	60.0% (15/25)	50.0% (9/18)	n.s. (*p* = 0.476)^d^	—
C/D	44.2% (19/43)	40.0% (10/25)	50.0% (9/18)		
TNM Dulguerov & Calcaterra					
T1/2	58.1% (25/43)	64.0% (16/25)	50.0% (9/18)	n.s. (*p* = 0.476)^d^	—
T3/4	41.9% (18/43)	36.0% (9/25)	50.0% (9/18)		
Mod. TNM Koka					
T1/2	51.16% (22/43)	52.0% (14/25)	44.4% (8/18)	n.s. (*p* = 0.356)^d^	—
T3/4	48.8% (21/43)	48.0% (11/25)	55.6% (10/18)		
TNM Biller/Resto					
T1	44.2% (19/43)	56.0% (14/25)	27.8% (5/18)	n.s. (*p* = 0.162)^d^	—
T2	51.2% (22/43)	44.0% (11/25)	72.2% (13/18)		
T3	4.7% (2/43)	56.0% (14/25)	27.8% (5/18)		
T1 vs. T2 vs. T3	44.2% (19/42)	40.0% (10/25)	66.7% (12/18)	n.s. (*p* = 0.167)^d^	
T2–3 or T2–4	55.8% (24/43)	4% (1/25)	5.6% (1/18)		
Hyam's grading dichotomized	(available *n* = 40/43)	(available *n* = 23/25)	(available *n* = 17/18)	***p* = 0.002** ^d^	***p* = 0.005**
I–II (*n*, %)	53.5% (23/43) 57.5% (23/40)^e^	72% (18/25) 78.3% (18/23)^e^	27.8% (5/18) 29.4% (5/17)^e^		
III–IV (*n*, %)	39.5% (17/43) 42.5% (17/40)^e^	20% (5/25) 21.7% (5/23)^e^	66.7% (12/18) 70.6% (12/17)^e^		
Ki‐67‐Index values (*n*, %)	13.4 ± 1.8 (M 10.15, R 0.26–40.2)	8.3 ± 1.7 (M 5.27, R 0.26–24.5)	19.8 ± 2.7 (M 16.3; R 5.1–40.2)	***p* = 0.001** ^a^	**—**
Ki‐67‐Index dichotomized^c^	(available *n* = 36/43)	(available *n* = 20/25)	(available *n* = 16/18)	***p* = 0.004** ^d^	***p* = 0.009**
< 10% (*n*, %)	34.9% (15/43) 41.7% (21/36)^f^	52.0% (13/25) 65.0% (13/20)^f^	11.1% (2/18) 12.5% (2/16)^f^		
≥ 10% (*n*, %)	48.8% (21/43) 58.3% (21/36)^f^	28% (7/25) 35.0% (7/20)^f^	77.8% (14/18) 87.5% (14/16)^f^		
Surgery only (*n*, %)	25.6% (11/43)	24.0% (6/25)	27.8% (5/18)	n.s. (*p* = 0.356)^d^	—
Resection status/margins (*n*, %)					
Negative	72.1% (31/43)	80.0% (20/25)	61.1% (11/18)	n.s. (*p* = 0.086)^d^	—
Unknown/close/positive	27.9% (12/43)	20.0% (5/25)	38.9% (7/18)		
Post‐OP RT (*n*, %)	67.4% (31/46)	76.0% (19/25)	66.7% (12/18)	n.s. (*p* = 0.137)^d^	—
Post‐OP RT‐dose (Gy)	57.8 ± 1.7 (M 60.0; R 24–68.4)	55.1 ± 2.6 (M 57.6; R 24–68.4)	62.10 ± 1.15 (M 62.1; R 55–66.6)	—	—
Combined therapy (S + RT ± ChT; *n*, %)	74.4% (32/43)	76.0% (19/25)	72.2% (13/18)	n.s. (*p* = 0.203)^d^	— —
Time to first treatment failure (TPR, *n*, %)	—	—	63.6 ± 17.3 (M 73.2; R 1–268)	—	—
Time to first recurrence (*n*, %)	—	—	67.3 ± 25.3 (M 107.5; R 5–268)	—	—
TPF‐free time (months: mean, median, range)	132.9 ± 21.77 (M 142.8; R 1–494)	182.8 ± 32.1 (M 119.0; R 8–494)	63.6 ± 17.3 (M 73.2; R 1–268)	***p* = 0.005** ^a^	—
Overall crude survival time (months)	162.3 ± 21.54 (M 122.0; R 4–494)	182.8 ± 32.1 (M 119.0; R 8–494)	133.9 ± 25.3 (M 123.5; R 4–309)	n.s. (0.489)^a^	—
Crude survival (OS):					
Alive	58.1% (25/43)	60.0% (15/25)	55.6% (10/18)	n.s. (*p* = 1.0)^b^	—
Dead	41.9% (21/43)	40.0% (10/25)	44.4% (8/18)		
Crude survival: last status					
AND	46.5% (20/43)	60.0% (15/25)	27.8% (5/18)	***p* = 0.0001** ^b^	—
AWD	11.6% (5/43)	0%	27.8% (5/18)		
DAD^b^, ^g^	27.9% (12/43)	40.0% (10/25)	11.1% (2/18)		
DOD	14.0% (6/43)	0%	33.3% (6/18)		
Crude survival: OS	58.1% (25/43)	60% (15/25)	55.6% (10/18)	n.s. (*p* = 1.00)^b^	
Crude survival: DSS	86.0% (37/43)	100% (25/25)	66.7% (12/18)	***p* = 0.003** ^b^	—
30‐years DSS according to Kaplan–Meier	84.9%	100%	66.7%	***p* = 0.003** ^d^	—
30‐years OS according to Kaplan–Meier	43.3%	44.2%	48.9%	n.s. (*p* = 0.576)^d^	—
30‐years TPRFS/DFS according to Kaplan–Meier	44.3%	—	—	—	—

Abbreviations: AND, alive, no disease; AWD, alive with disease; c/p, clinical/pathological; ChT, chemotherapy; CRT, chemoradiotherapy; DAD, dead due to another disease; DOD, dead of disease; DSS, disease‐specific survival; M, median; n.d., no data/not calculated; n.s., not significant; O, other (therapy); R, range; RT, radiotherapy; S, surgery; TNM, primary tumor, regional nodes, metastasis (staging); TPR, tumor progression/recurrence.

^a^ Mann–Whitney *U* test.

^b^ Chi‐Square test with two‐sided exact significance.

^c^ Dichotomized Ki‐67 LI was used for cox regression analysis.

^d^ Log‐rank‐test.

^e^ Hyam's grading in 93% of all cases (40/43) available.

^f^ Ki‐67 index in 83.72% of all cases (36/43) available.

^g^ Both patients died due to another disease (DAD) and with persistent disease (DWD).

Therapy was carried out with curative intent in all of the patients. A bicoronal/transcranial approach was used in 58.1% (25/43), a transfacial approach in 7% (3/43), and endoscopic resection in 34.9% (15/43). Negative margins were obtained in 72.1%. Combined therapy was conducted in 74.4% and post‐operative RT in 67.4%. The mean radiation dosage was 57.8 ± 1.7Gy.

Hyams grading could be assessed in 93% of the patients (40/43; grade I 20%, 8/40; grade II 37.5%, 15/40; grade III 40%, 16/40; grade IV: 2.5%, 1/40). High‐grade tumors were present in 42.5% (17/40).

Ki‐67 LI could be assessed in 83.7% of the patients (36/43). The mean value was 13.4 ± 1.8%; in 58.3%, the Ki‐67 LI was ≥ 10%. A significant association between the categories of Hyams grade and the Ki‐67 LI was noted (*p* = 0.019).

In 3 cases in whom no Hyams‐grading and no Ki‐67 could be determined and 4 cases in whom only Hyams‐grading could be measured, but not the Ki‐67 LI, no or not sufficient amount of tissue was available. Two of these cases developed TPR (for details see Table S1).

TP after curative therapy was observed in one case before the end of the first month; the patient survived only 4 months and died due to extensive intracranial tumor growth (tumor stage Kadish/Morita C, Dulguerov&Calcaterra T4, Hyams‐grade 3, Ki‐67 LI 26.5%). TR was diagnosed in 39.5% of the patients (17/43) after a mean of 67.3 ± 25.3 months (M 73.7, R 5–268). Local recurrences were observed in 16.3% (7/43), regional recurrences in 11.6% (5/43), distant recurrences in 7% (3/43), loco‐regional in 4.7% (2/43) and regional and distant recurrences in 2.3% (1/46). Altogether, primary treatment failure (TPR) was observed in 41.9% (18/43) after a mean of 63.6 ± 17.3 months (M 73.2, R 1–268). Further details are included in the Table S1.

The 30‐years overall survival (OS), disease‐specific survival (DSS) and TPRFS/DFS in the Kaplan–Meier analysis were 43.3%, 84.9%, and 44.3% for all patients (Table 2).

### 3.2 Group Analysis of Patients Without and With TPR

Patients without (*n* = 25) or with TPR (*n* = 18) were compared with regard to prognostic factors (Table 2).

### 3.3 Univariate Analysis to Investigate the Association/Correlation of Parameters With TPR and TPRFS

**FIGURE 1:**
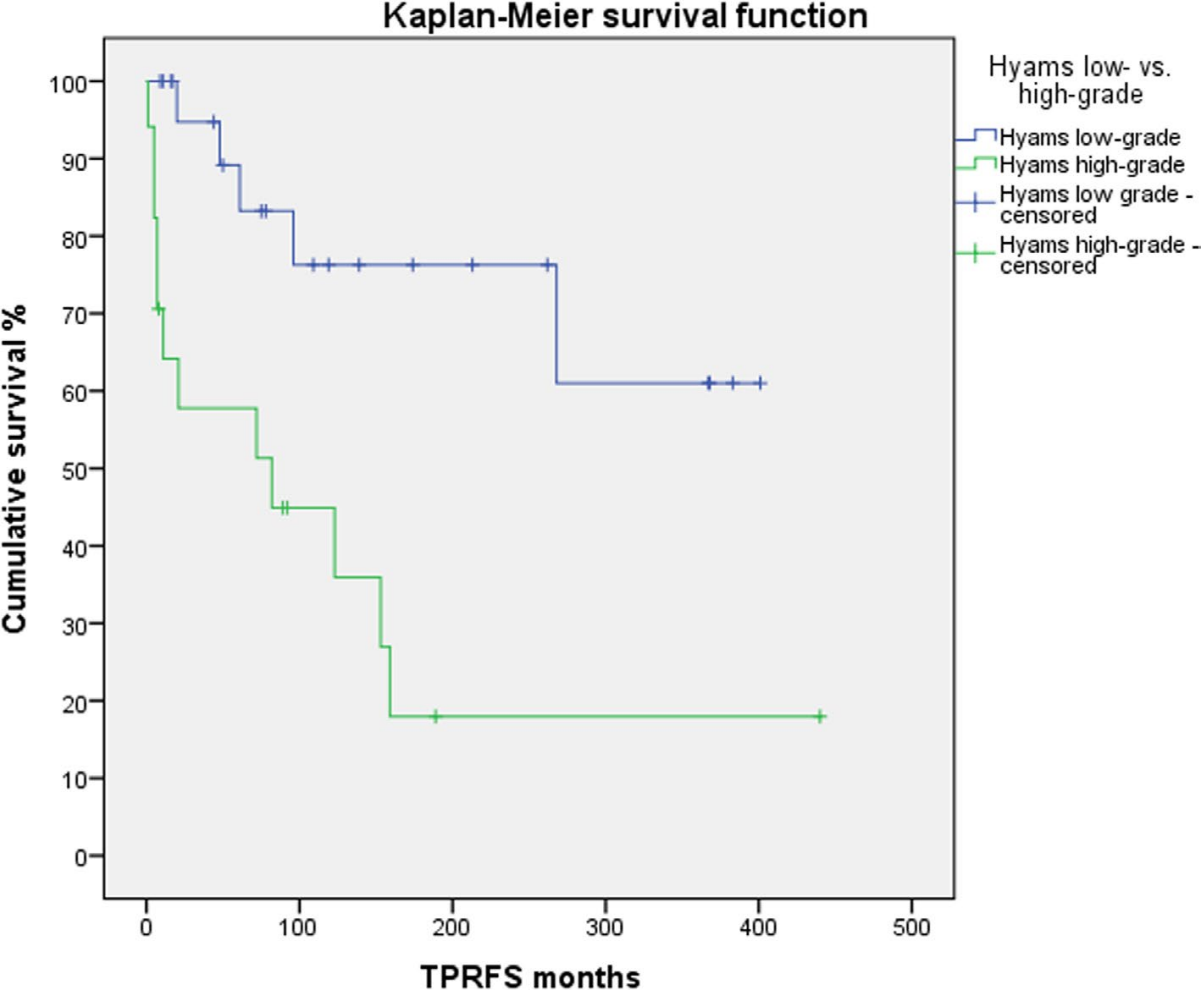
Primary progression‐free and recurrence‐free survival (TPRFS) among patients with low‐grade tumors (blue line) vs. those with high‐grade tumors (green line; log‐rank test *p* = 0.002). [Color figure can be viewed in the online issue, which is available at www.laryngoscope.com.]

**FIGURE 2:**
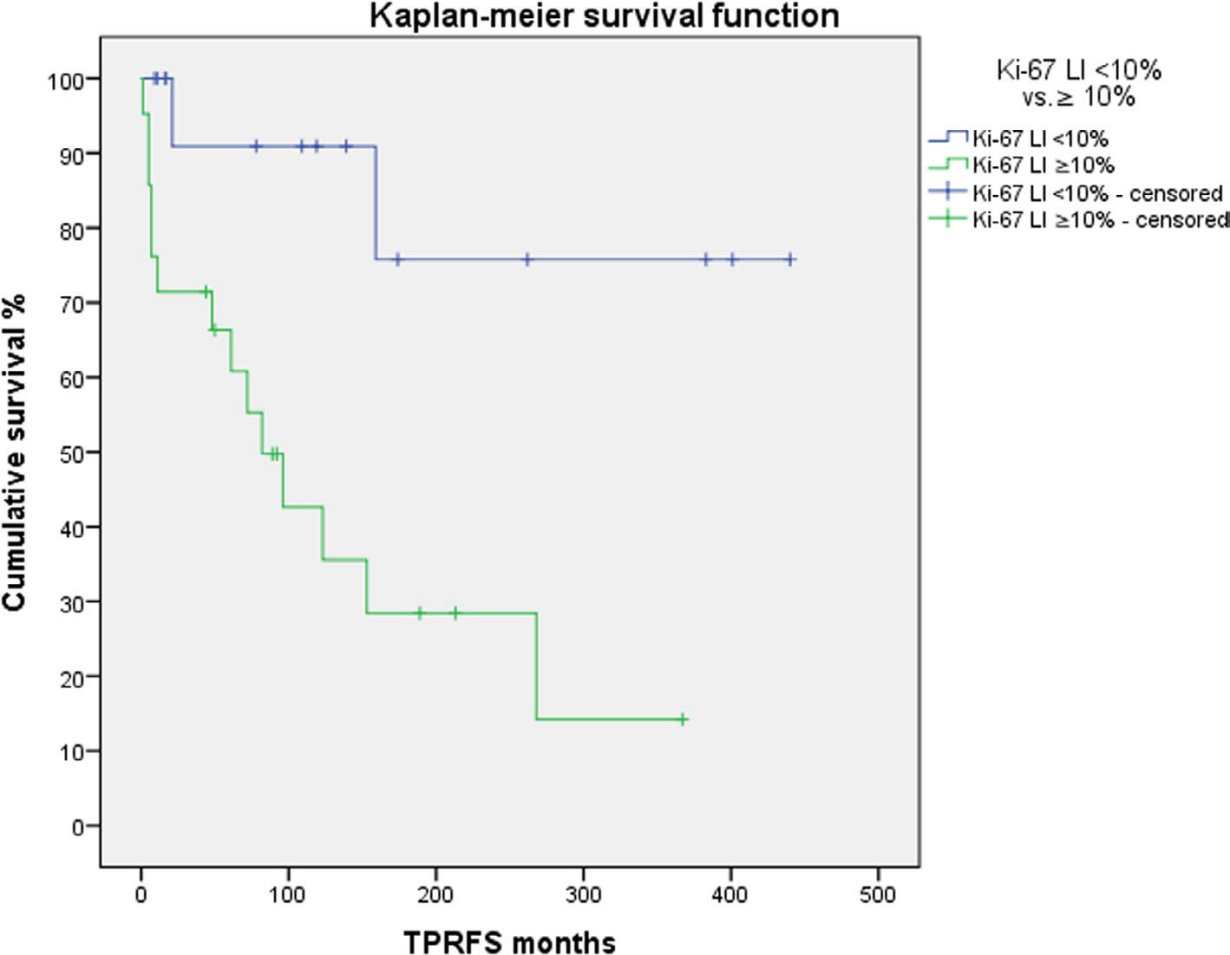
Primary progression‐free and recurrence‐free survival (TPRFS) among patients with Ki‐67 < 10% (blue line) vs. those with Ki‐67 ≥ 10% (green line; log‐rank test *p* = 0.004). [Color figure can be viewed in the online issue, which is available at www.laryngoscope.com.]

Younger age (*p =* 0.032) and higher Ki‐67 values (*p =* 0.001) were significantly associated with a shorter time to TPR. TPRFS according to Kaplan–Meier was significantly worse for high‐grade tumors (*p =* 0.002, Figure 1) and tumors showing a Ki‐67 LI ≥ 10% (*p =* 0.004, Figure 2).

**FIGURE 3:**
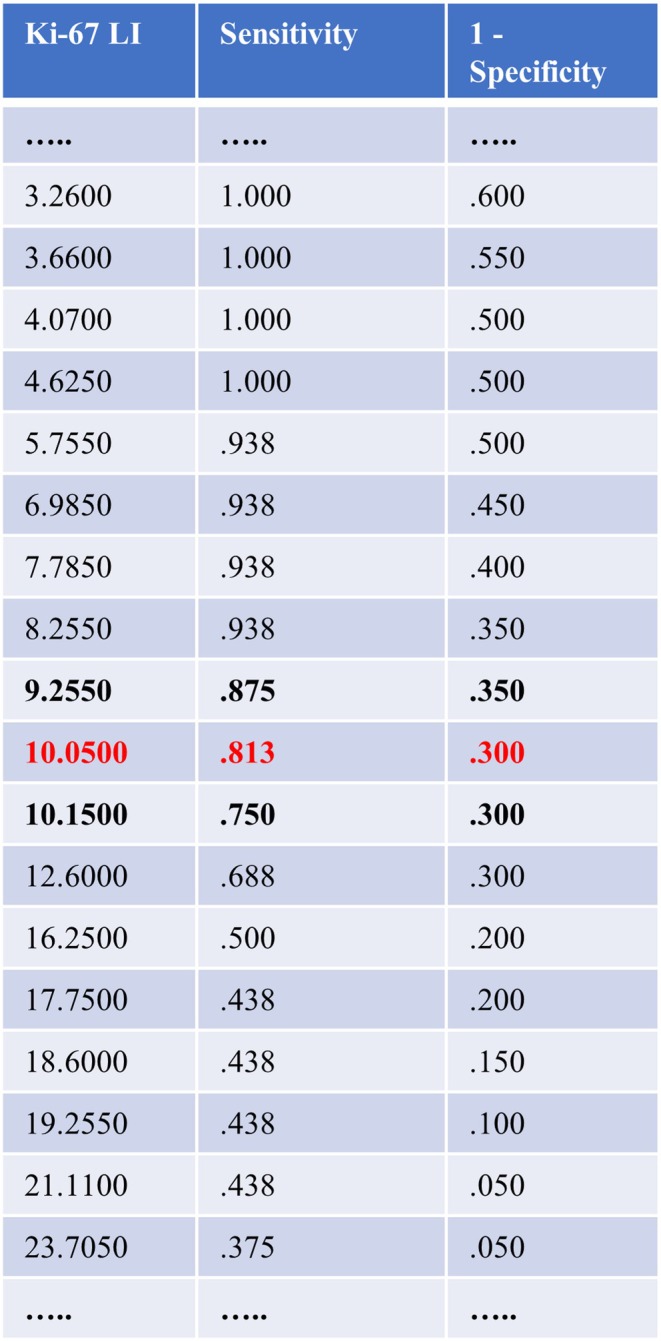
Ki‐67 labeling index values for Ki‐67 labeling index values (%) relative to the event of tumor progression/recurrence (TPR). The cut‐off value is marked with red letters. [Color figure can be viewed in the online issue, which is available at www.laryngoscope.com.]

**FIGURE 4:**
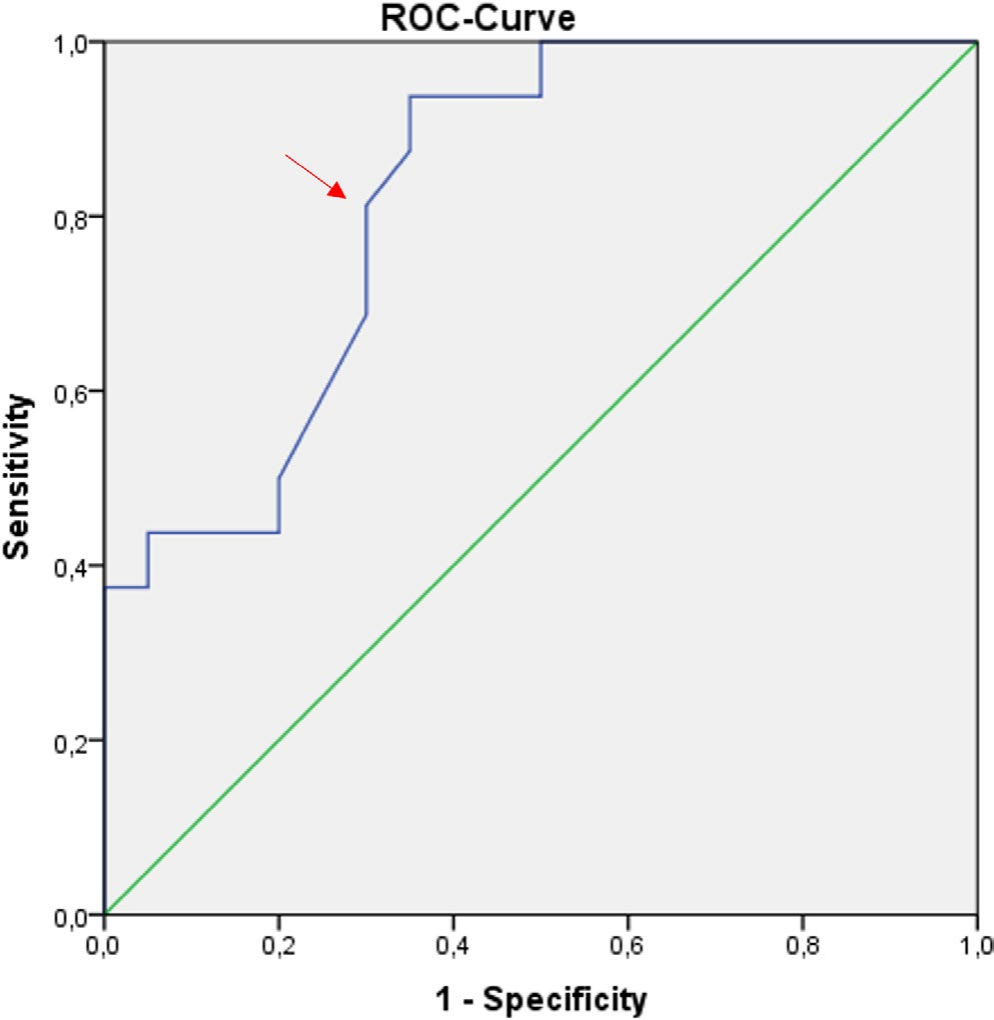
Receiver operating characteristics (ROC) curve for Ki‐67 labeling index values (%) relative to the event of tumor progression/recurrence (TPR). The area under the curve is 0.83. The cut‐off point is marked with a red arrow. [Color figure can be viewed in the online issue, which is available at www.laryngoscope.com.]

The cut‐off value for the Ki‐67 LI in the ROC curve showed the optimal ratio between sensitivity and 1‐specificity of TPR at 10.1%, which was the final cut‐off value, with an area under the curve of 0.83 (Figures 3 and 4).

**FIGURE 5:**
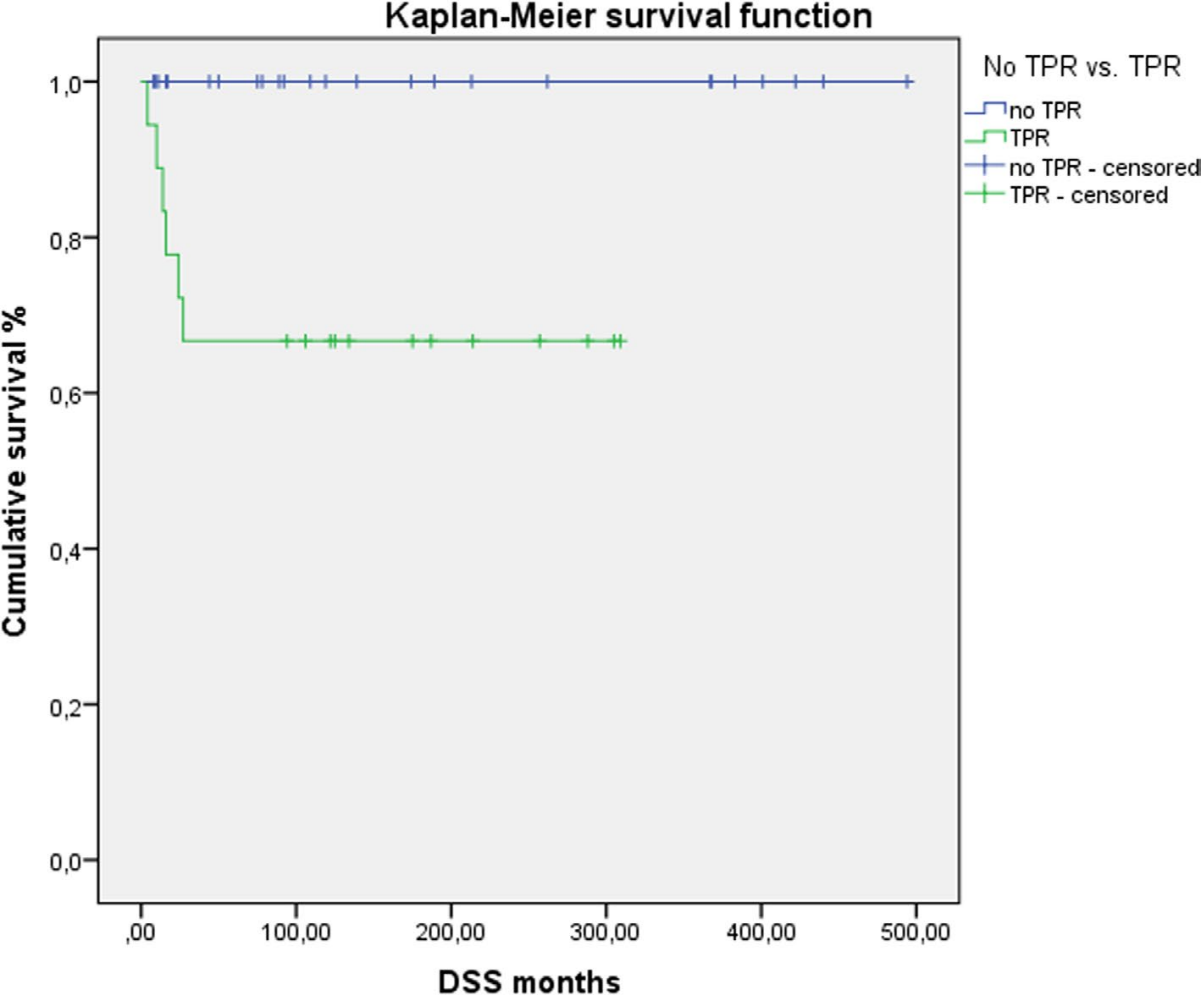
Kaplan–Meier 30‐years disease‐specific survival (DSS) among patients without tumor progression/recurrence (TPR, *n* = 25) and with TPR (*n* = 18; log‐rank test *p* = 0.003). [Color figure can be viewed in the online issue, which is available at www.laryngoscope.com.]

For patients without TPR, the state of crude survival and crude DSS at last presentation was significantly better (*p* = 0.0001; *p* = 0.003), the TPR‐free survival time was significantly longer (182.76 vs. 63.6 months; *p =* 0.005), but the crude total survival time showed no significant differences (182.76 vs. 133.9 months; *p =* 0.489). The 30‐years DSS in the Kaplan–Meier analysis was 100% for patients without TPR and 66.7% for patients with TPR (*p =* 0.003, Figure 5; Table 2).

### 3.4 Multivariate Regression Analysis to Investigate the Association/Correlation of Parameters With TPR and TPRFS

For Cox regression analysis, age, dichotomized Hyams‐grade, and Ki‐67 LI categories were used. TPRFS was significantly poorer in younger patients (*p* = 0.033, HR 0.9, 95% CI 0.9–0.1), in tumors with Hyams grade III–IV (*p =* 0.005, HR 7.2, 95% CI 1.8–29.2) and with a Ki‐67 LI of ≥ 10% (*p =* 0.009; HR 9.4, 95% CI 1.7–51.5; Table 2). To reduce a possible bias due to a significant correlation of Hyams grading and the Ki‐67 LI, Cox regression analysis was performed with one factor excluded: it revealed that higher Ki‐67 categories were significantly associated with worse TPRFS (*p* = 0.010, HR 7.2, 95% CI 1.6–32.5), if Hyams grading was excluded. Vice versa, Hyams grading III–IV was significantly associated with worse TPRFS (*p* = 0.004, HR 4.8, 95% CI 1.6–13.9) if Ki‐67 LI was excluded.

## 4 Discussion

All 43 patients included in this study were treated with curative intent in our department, according to published standards [24, 35, 44] and treatment strategies (Tables 1 and 2) [4, 5, 7, 8, 9, 10, 11, 12, 13, 14, 15, 16, 17, 18, 19, 20, 21]. Identifying risk factors for TPR could help suggest ways in which patients with tumors that are at high risk for primary treatment failure can be recognized earlier [6, 64].

The development of TP despite curative therapy has been reported with a frequency of 5.6%–28.6% in a few publications [29, 34, 53, 54, 55, 56] and in case reports [57, 58], with only a few details provided. TP was in nearly all cases associated with a short survival time, similar to our reported case, who survived only 4 months.

TR have been reported to be significantly associated with high‐grade tumors (Hyams‐grade III–IV) [34, 44, 45, 66], advanced tumor‐stage [45, 64, 66, 68, 70], expression of the biomarker INSM1 [68], and a reduced survival time [18, 45, 66, 69].

Younger age (*p =* 0.033, HR 0.9) was found to be weakly significantly associated with a poorer TPRFS, which is in contrast to the literature results, which suggest that age > 50–65 years is a negative prognostic factor [9, 22, 23, 24, 25, 26, 35]. The differing, weakly significant results may be explicable by potential referral bias. The present results also revealed that age < 50 years compared to ≥ 50 years did not have a significant impact on TPRFS—supporting the referral bias explanation and simultaneously indicating the effectiveness of the salvage therapy performed, which is not within the scope of this manuscript.

Hyams‐grade has been reported to be associated with TPR, a poorer survival and outcome in ONB [11, 23, 28, 33, 34, 35, 36, 37, 38, 39, 40, 41, 42, 43, 44, 45, 52]. In a recent systematic review (including 205 studies and 965 patients), after multivariate analysis Hyams‐grade of III–IV was associated with poorer 5‐ and 10‐years survival [35]. In a meta‐analysis (including 33 studies and 492 patients), it was found that recurrence‐free survival was significantly poorer in high‐grade tumors [44]. In one report, Kaur et al. stated that the 2‐ and 5‐years progression‐free survival was significantly lower in patients with high‐grade tumors (86% vs. 65% and 73% vs. 49%). It was concluded that Hyams‐grading “appeared to be the best way of predicting the prognosis and for selecting patients for adjuvant therapy.” [36], which was also concluded in another report [23]. In both reports, conventional tumor‐stage classifications were not considered to be adequate for assessing the biological behavior of ONB and Hyams‐grade was added. In a recent multicenter analysis, conventional tumor‐staging systems were each stratified according to low and high Hyams‐grade, resulting in a maximum of eight stages. The HR for disease progression was highest in higher tumor‐stages, combined into Hyams‐grade III–IV. It was concluded that incorporating the Hyams‐grading into the known tumor staging classifications for ONB “is associated with improved estimation of disease progression.” [52] In accordance to this, in the present study the distribution of tumor‐stages did not significantly differ between patients without or with TPR (Table 2), but high‐grade tumors were significantly positively associated with a poorer TPRFS (*p* = 0.005, HR 7.2; Table 2).

Two studies have reported a significant positive association between Hyams grades and Ki‐67 LI values [47, 48], as was also found in the present study (*p* = 0.019). Ki‐67 LI grading has been proposed for neuroendocrine tumors (grade 1: ≤ 2%, grade 2: 3–20%, grade 3: ≥ 20%) using the World Health Organization criteria [72, 73]. The dichotomized Ki‐67 LI used in the present study (< 10% vs. ≥ 10%) may correspond to grades 1–2 vs. 2–3. A possible correlation between the Ki‐67 LI and survival has been described in some publications [46, 47, 48]. In the present study, higher Ki‐67 values were significantly associated with shorter time to TPR (*p* = 0.001) and a Ki‐67 LI of ≥ 10% (*p* = 0.009) associated with a significantly worse TPRFS. A Ki‐67 LI cut‐off value of ≥ 25% has been reported to be significantly associated with poorer survival in other reports [47, 48]. In the present study, the mean value for all patients was 13.4% (range 0.26–40.2) and patients with TPR had a mean of 19.8% (range 4.8–40.2). The range of values (1–93) was higher in the report by Classe et al. [48], but the optimal values in the ROC curve (maximum threshold level of 0.75 vs. 0.81) were comparable with the present findings. The Ki‐67 cut‐off value between 22% and 27% published there was higher compared to our results (9.25%–10.2%, Figures 3 and 4). Despite these differences, the data point to Ki‐67 LI as a prognostic marker.

Hyams‐grade and Ki‐67 LI values can both be assessed with an adequate preoperative transnasal biopsy and/or postoperative analysis of the surgical specimen. If a high‐grade ONB with a Ki‐67 LI of ≥ 10% is diagnosed, then management could be adapted in order to reduce the risk for TPR and increase the TPRFS. In this context, the importance of histopathologic prognostic factors was highlighted in further reports. In one, two of three cases of recurrence were associated with Hyams‐grade III and it was stated that “Chemotherapy seems warranted for high‐grade tumors (Hyams III–IV),…” [64] In another, 78.6% of all and 71.4% of all regional recurrences were associated with high‐grade tumors. It was stated that the “non‐negligible incidence of regional recurrences, partly in unusual localizations, leads us to consider the need to identify the ‘recurrence‐friendly’ cases and to perform individualized elective irradiation of the neck in cases with high‐risk features.” [6] In the present study, 70.5% of patients with TPR, 66.7% with recurrences and 75% (six of eight) of those with regional recurrences had high‐grade tumors. For a Ki‐67 LI ≥ 10%, the rate was 87.5% for all, 86.7% for all recurrences and 100% for regional recurrences (Table S1). Our results published here suggest that Hyams‐grade III–IV and a Ki‐67 LI > 10% may be used to define high‐risk tumors. Significances could be observed also after Cox regression analysis was performed with one factor excluded. Higher Ki‐67 LI categories (*p* = 0.01, HR 7.2) or Hyams grading III–IV (*p* = 0.004, HR 4.8) were each significantly associated with worse TPRFS reducing a possible bias.

Improved and adapted, perhaps more aggressive, primary treatment strategies taking into account the prognostic factors addressed in this manuscript could have a favorable impact on TPRFS and may spare additional salvage therapies to at least a part of the patients. Differences in the current survival state and 30 years DSS according to Kaplan–Meier point to possible unfavorable long‐term effects after the occurrence of TPR (Table S1).

This study has limitations. It was a retrospective analysis over 40 years of patients treated in a single institution, with relatively low case numbers and potential referral bias. Although no differences were noted in comparison with the literature, institutional bias involving the treatment strategies cannot be excluded. Also, changes in the treatment regime in the long‐term course due to new developments may have had influence on the results.

## 5 Conclusion

Among the relevant prognostic factors, younger age and higher Ki‐67 LI values were significantly associated with a shorter time to TPR. In particular, patients with high‐grade tumors and a Ki‐67 LI ≥ 10% had a significantly poorer TPRFS (*p* = 0.005; *p* = 0.009). A possible impact on long‐term survival is indicated by the 30‐years DSS, which for cases with TPR also was significantly worse in comparison with cases without TPR (67% vs. 100%, *p* = 0.003). As both histopathologic factors can be measured preoperatively, it is also possible to assess these very early in order to adapt the treatment.

## Conflicts of Interest

The authors declare no conflicts of interest.

## Supporting information


**Table S1.** Data of patients with olfactory neuroblastoma after development of TPR (*n* = 18): Tumor stage, histopathologic factors, first therapy, tumor progression/tumor recurrence (TPR), salvage therapy after primary therapy failure; current survival state, crude total survival time.
