# Supplementary material for: Hyams Grade and Ki‐67 as Predictive Factors for Primary Treatment Failure in Olfactory Neuroblastoma
Source: Laryngoscope. 2025 May 9;135(10):3691–702. doi: 10.1002/lary.32238 (PMC12475542; doi:10.1002/lary.32238)
Supplement: Supplementary file 1 — Table S1. Data of patients with olfactory neuroblastoma after development of TPR (n = 18): Tumor stage, histopathologic factors, first therapy, tumor progression/tumor recurrence (TPR), salvage therapy after primary therapy failure; current survival state, crude total survival time. [file LARY-135-3691-s001.docx]

**Supplemental Table:** Data of patients with olfactory neuroblastoma after development of TPR (n=18): Tumor stage, histopathologic factors, first therapy, tumor progression/tumor recurrence (TPR), salvage therapy after primary therapy failure; current survival state, crude total survival time.

| **Parameter**  **Patient**  **number;**  **(age, gender, year of first diagnosis)** | **Tumor stage**  Kadish/Morita;  Dulguerov&  Calcaterra | **Histopathology**  Hyams-grade;  Ki-67 LI (%) | **Therapy**  **Modality**  Surgery (resection state) + AT | **Therapy failure/TPR**  Location;  Time after  first therapy (months) | **First salvage therapy**  Yes vs. no;  **Success**  yes vs. no  (surgery R-status) | **Current**  **survival**  **state** | **Crude total survival time (months)** |
| --- | --- | --- | --- | --- | --- | --- | --- |
| **1**;  (59, f, 1984) | B;  T2 | n.d.;  n.d. | S (R0) | TP local;  6 | Yes;  Yes (S, R1 + RT) | DOD | 10 |
| **2**;  (32, m, 1985) | C;  T3 | 3;  8.5 | S (R1) + RT | TR local;  21 | Yes;  No (S, R2) | DOD | 27 |
| **3**;  (59, f, 1985) | C;  T4 | 2;  n.d. | S (R1) +RT | TR local;  20 | Yes;  No (S, R1) | DAD/  DWD | 94 |
| **4**;  (50, f, 1985) | B;  T2 | 3;  17.5 | S (Rx) | TR local+regional (bilateral);  5 | No;  ChT ~ | DOD | 14 |
| **5**;  (77, f, 1988) | B;  T1 | 4;  22.9 | S (R1) | TR local;  5 | Yes;  RT | DOD | 16 |
| **6**;  (50, m, 1988 | C;  T4 | 3;  26.5 | S (R1) | TP Local;  1 | No;  ----- | DOD | 4 |
| **7**;  (38, f, 1996) | B;  T2 | 2;  10.2 | S (Rx) + RT | TR regional;  96 | Yes;  Yes (ND + RT) | AND | 309 |
| **8**;  (34, f, 1997) | C;  T3 | 3;  35.0 | S (R0) + RT | TR regional (bilateral);  7 | Yes;  RCT | AND | 288 |
| **9***;*  (31, f, 1997) | B;  T2 | 2;  15.0 | S (R0) + RT | TR distant (meningeal);  268 | Yes;  Yes (S, Rx, +SRT) | AWD | 305 |
| **10**;  (51, m, 1999) | B;  T2 | 3;  5.1 | S (R0) + RT | TR local;  159 | Yes;  Yes (S, R0 + RT) | AND | 257 |
| **11**;  (50, m, 2003 | C;  T3 | 3;  25.0 | RT + S (R0) + ChT | TR local+regional; 123 | Yes (RN + S, R2 + ND + ChT) + | DAD/  DWD | 214 |
| **12**;  (68, m, 2004) | C;  T3 | 3;  40.2 | S (Rx) + RT | TR distant (meningeal);  11 | Yes;  Yes (S, Rx + RT +SRT) | DOD | 24 |
| **13**;  (27, m, 2007) | B;  T2 | 2;  15.0 | S (R0) + RT | TR regional;  61 | Yes;  Yes (ND + RT) | AND | 175 |
| **14**;  (58, f, 2006) | B;  T2 | 3;  10.1 | S (R0) + RCT | TR regional;  153 | Yes;  Yes (ND + RCT) | AND | 187 |
| **15**;  (46, f, 2011) | C;  T3 | 2;  10.0 | S (R0) # | TR local;  48 | Yes;  RT | AWD | 134 |
| **16**;  (47, m, 2012) | B;  T2 | 3;  25.0 | S (R0) + RT | TR regional + distant;  7 | Yes;  Yes (ND + RCT + AB) | AWD | 122 |
| **17**;  (43, f, 2013) | C;  T3 | 3;  15.0 | S (R0) + RT | TR distant (spine); 82 | Yes;  Yes (S, Rx + RCT + SRT + AB) | AWD | 106 |
| **18**;  (45, m, 2013) | C;  T3 | 3;  35.0 | S (R0) + RT | TR regional;  72 | Yes;  Yes (ND + SRT + RCT) | AWD | 125 |

TPR, tumor progression and/or recurrence; S, surgery; AT, adjuvant therapy; RT, radiotherapy; RCT, radio-chemotherapy; ChT, chemotherapy; STR, stereotactic radiotherapy; ND, neck dissection; AB, therapy with antibodies; RN, therapy with radionuclides; R0, negative margins; R1, positive microscopic margins; R2, positive macroscopic margins; Rx, margins not clearly defined;

n.d.= no data

~ RCT planned, but due to rapid tumor growth of recurrence only palliative ChT

+ RT planned, but due to bad general condition of the patient not conducted

# RCT planned, but refused by the patient
